# Psychological outcomes in paediatric major trauma patients who require invasive management: protocol for a systematic review and meta-analysis

**DOI:** 10.1136/bmjopen-2025-101971

**Published:** 2025-08-19

**Authors:** Owen Hibberd, Caroline E Thomas, Sarah Gentle, Sandi Angus, Spyridon Karageorgos, Veronica Phillips, Stephen H Thomas

**Affiliations:** 1 Emergency and Urgent Care Research in Cambridge (EURECA), PACE Section, Department of Medicine, Cambridge University, Cambridge, UK; 2 Blizard Institute, Queen Mary University of London, London, UK; 3 Georgetown University, Washington, Washington, DC, USA; 4 National and Kapodistrian University of Athens, Athens, Greece; 5 University of Cambridge Medical Library, Cambridge, UK; 6 Department of Emergency Medicine, Harvard Medical School, Boston, Massachusetts, USA

**Keywords:** Paediatric intensive & critical care, ACCIDENT & EMERGENCY MEDICINE, TRAUMA MANAGEMENT, Paediatric orthopaedic & trauma surgery, Psychological Stress

## Abstract

**Abstract:**

**Introduction:**

Paediatric major trauma patients with more severe injuries and physiological or biochemical abnormalities as a result of the injury are more likely to require invasive management in the form of an operation/interventional radiology (IR). Adverse psychological outcomes, such as post-traumatic stress disorder, anxiety, depression and adjustment disorder, are frequently observed in paediatric patients with major trauma. Similarly, it is recognised that children and adolescents who have invasive management are also at an increased risk of adverse psychological outcomes. However, it is not known to what extent major trauma patients requiring invasive management are at risk of adverse psychological outcomes compared with those managed conservatively. This study aims to determine whether paediatric major trauma patients who require an operation/IR have increased odds of having an adverse psychological outcome compared with those who are managed conservatively.

**Methods and analysis:**

The Preferred Reporting Items for Systematic Review and Meta-Analysis Protocols guidelines will be used to construct this review. The databases Medline (via Ovid), Embase (via Ovid), PsycInfo (via Ebscohost) and Cinahl (via Ebscohost) will be searched from inception to February 2025. Both title and abstract screening and full-text screening will be done by two reviewers, with an adjudicating third reviewer. For randomised controlled trials, the Cochrane Risk of Bias Tool will be employed, while for non-randomised studies, the Newcastle-Ottawa Quality Assessment Scale will be used. We will assess bias using contoured funnel plots (with p set at 0.01, 0.05 and 0.10), non-parametric trim-fill analysis, leave-one-out analysis and Galbraith plotting. We will execute formal (Egger) testing for funnel plot asymmetry and also calculate prediction intervals if sufficient study N of 10 is accrued. Certainty and confidence in cumulative evidence will be evaluated using the Grading of Recommendations Assessment, Development and Evaluation (GRADE) approach.

**Ethics and dissemination:**

Ethical review is not required as no original data will be collected. Results will be disseminated through peer-reviewed publications and at academic conferences.

**PROSPERO registration number:**

CRD42025643459.

**STRENGTHS AND LIMITATIONS OF THIS STUDY**

- The protocol follows the Preferred Reporting Items for Systematic Review and Meta-Analysis Protocols guidelines.
- The search algorithm is open with wide inclusion criteria, allowing identification of a wide range of papers to best inform the reviews' conclusions.
- Each stage will involve adjudication by two independent reviewers for screening, data extraction and quality assessment.
- The heterogeneity of the included studies may limit the ability for quantitative summary in the form of meta-analysis, with a qualitative summary anticipated for a wide section of the literature.

## Background and rationale

 Major trauma remains a leading cause of morbidity and mortality in children and adolescents.^1 2^ Paediatric major trauma patients with more severe injuries and physiological or biochemical abnormalities as a result of the injury are more likely to require invasive management in the form of an operation/interventional radiology (IR).^3^,^6^ In addition to physical injuries requiring intervention, holistic care of children and adolescents who have had a serious injury should also consider their mental health needs.^7 8^ However, integrated mental healthcare alongside the treatment of the physical injuries is not universally seen.^9^

Adverse psychological outcomes, such as post-traumatic stress disorder (PTSD), anxiety, depression and adjustment disorder, are frequently observed in paediatric patients with major trauma.^7 10 11^ Injured children and adolescents may also be at a higher risk for developing adverse psychological outcomes compared with their adult counterparts.^10^,^14^ Admission to the paediatric intensive care unit (PICU) is also recognised to be associated with adverse psychological outcomes and often goes hand-in-hand with the management of major trauma.^11 15 16^ Similarly, it is recognised that children and adolescents who have invasive management are also at an increased risk of adverse psychological outcomes.^17 18^ However, it is not known to what extent major trauma patients requiring invasive management are at risk of adverse psychological outcomes compared with those managed conservatively.

### Aims

This study aims to determine whether paediatric major trauma patients who require an operation/IR have increased odds of having an adverse psychological outcome compared with those who are managed conservatively. By identifying paediatric major trauma patients at increased risk of adverse psychological outcomes, we can enhance potential screening for psychological trauma, help pinpoint those most at risk of adverse outcomes and offer early targeted holistic support for this group of patients.

## Methods

### Eligibility criteria

Population, intervention, comparison, outcomes and study design criteria are detailed in table 1. Inclusion criteria will include all clinical trials, observational studies and case series with data which facilitates the comparison of the odds of a new psychological disorder (eg, PTSD/anxiety/depression or adjustment disorder) in paediatric major trauma patients (defined as injury severity score >15 and <16 years) who underwent invasive management (operation/IR) compared with those who were conservatively managed. Studies published in any language that meet the inclusion criteria will be included. If the study is published in a language other than English, it will be included if sufficient data can be extracted using open-access translation software such as *Google Translate*.

**Table 1:** . Inclusion and Exclusion Criteria

PICOS strategy	Inclusion criteria	Exclusion criteria
P—population	Paediatric (<16 years) major trauma patients (defined as injury severity score>15)	Adult major trauma patients
I—intervention	Invasive management (operation/interventional radiology)	N/A
C—comparator	Conservative management	N/A
O—outcome	The diagnosis of a psychological disorder (eg, post-traumatic stress disorder/anxiety/depression or adjustment disorder)	N/A
S—study design	Clinical trials (randomised, quasi-randomised and non-randomised) Observational studies (cohort and case–controlled) Case series	Systematic reviews Narrative reviews Editorials/commentaries Opinion articles Case reports

N/A, not available.

### Information sources

#### Search strategy

The databases Medline (via Ovid), Embase (via Ovid), PsycInfo (via Ebscohost) and Cinahl (via Ebscohost) will be searched from inception to August 2025 by VP. The search strategy was peer-reviewed by two librarian colleagues of VP using the Peer Review of Electronic Search Strategies checklist^19^ and evaluated against the Preferred Reporting Items for Systematic Review and Meta-Analysis Statement (PRISMA-S) guidelines.^20^ Databases will be searched by VP separately, rather than multiple databases being searched on the same platform. The search syntax was adapted for each database and to account for variation between thesaurus terms/controlled vocabulary across each database. Results will be imported to Endnote 21^21^ by VP for deduplication using the method outlined by Bramer *et al*.^22^ The searches will all be rerun prior to submission in order to include any papers published between the initial searching and submission for peer review. Dates when searches were run will be indicated in the results table.

The search will also involve checking reference lists of retrieved articles, conference abstracts and online study results. The grey literature will be searched using Google Scholar and the Grey Matters tool.^23^ If data are incomplete, then corresponding authors will be contacted for additional information. Results for the provisional testing of the search strategy prior to starting the systematic review are shown in online supplemental appendix 1.

#### Study records

The planned start date for the review is 1 September 2025 and the planned end date is 1 December 2025. Rayyan, an online Systematic Review program, will facilitate the review process.^24^ Abstract screening will identify studies in which adverse psychological outcomes were recorded in paediatric major trauma patients, while full-text screening will identify which studies included whether the patient had invasive management (operation/IR).

As per Cochrane recommendations for two reviewers to assess at least 10% of the records to adjudicate inclusion, abstracts of studies obtained through the search strategy (as well as those from independent sources and grey literature searches) will be screened independently by two review authors to identify studies that meet the inclusion criteria outlined above.^25^ The full texts of these articles will then be retrieved and reviewed independently by two review authors against the stated inclusion and exclusion criteria. Any disagreements will be resolved through discussion with a third reviewer.

A standardised form will be used to extract data from the included studies for the purpose of collating evidence and assessing study quality. Two review authors will independently extract the data. Any discrepancies will be identified and resolved by a third author.

The following data items will be extracted and recorded in an Excel spreadsheet (Microsoft Excel for Mac, V.16.72, 2023)^26^:

1. Hospital setting.
2. Study type.
3. Country of treatment.
4. Cohort size.
5. Gender.
6. Age.
7. Type of injury.
8. Injury Severity Score (ISS).^27^
9. Prior mental health diagnosis.
10. Head injury.
11. Fatality at the scene.
12. Family member also injured.
13. Death of a family member.
14. Screening tools used for adverse psychological outcomes.
15. Incidence of adverse psychological outcomes.
16. Operation.
17. IR.
18. Length of stay.
19. PICU admission.
20. Definition of functional outcomes.
21. Incidence of poor functional outcomes (defined by any quantitative quality of life measure).

### Outcomes and prioritisation

The main outcome is the new diagnosis of psychological disorder within 6 months of the trauma incident. For this review, this is categorised as a new diagnosis of PTSD/anxiety/depression or adjustment disorder defined by any means.

The association between adverse psychological outcomes and

1. Length of stay.
2. PICU admission.
3. Poor functional outcomes (defined by any quantitative quality-of-life measure).

### Risk of bias and confidence in cumulative evidence

Studies will be assessed clinically and methodologically (study design, comparability, outcome ascertainment and risk of bias).

The risk of bias will be assessed for all included studies. For randomised controlled trials, the Cochrane Risk of Bias Tool will be employed, while for non-randomised studies, the Newcastle-Ottawa Quality Assessment Scale will be used.^28 29^ This will include an assessment of the impact of missing data on effect estimates. Although it is acknowledged that the likely heterogeneity we will encounter will reduce the information available from funnel plotting and associated trim-fill analysis, the literature does suggest some utility from these techniques when these conditions are encountered.^30^ We will assess bias using contoured funnel plots (with p set at 0.01, 0.05 and 0.10), non-parametric trim-fill analysis, leave-one-out analysis and Galbraith plotting. We will execute formal (Egger) testing for funnel plot asymmetry and also calculate prediction intervals if sufficient study N of 10 is accrued.^31^ Certainty and confidence in cumulative evidence will be evaluated using the GRADE approach.^32^

### Data synthesis

Data will be synthesised following PRISMA guidelines.^33^

A descriptive analysis will be conducted. The descriptive analysis will also include a description of injured patients with a previous mental health diagnosis to describe the overall cohort. If the studies have sufficiently homogeneous populations, exposure and outcome data will be pooled, and a meta-analysis will be performed to calculate summary measures of effect, illustrated using a forest plot. Operation and IR will be analysed separately, but if these cannot be separated in the data extraction, they will be combined as ‘invasive management’. A random-effects model will be employed to account for anticipated differences between studies. A meta-analysis will be undertaken for the primary outcome of a new diagnosis of a psychological disorder in major trauma patients who underwent invasive management (operation/IR) compared with those who were conservatively managed. Where possible, prespecified subgroup analysis will be undertaken for length of stay and poor functional outcomes (defined by any quantitative quality of life measure). As per standard recommendations from Cochrane, we will plan to execute subgroup-focused analyses, such as meta-regression, if we can accumulate approximately 10 studies.^34^ If there is sufficient data, a meta-regression will include other potential effect modifiers such as ISS, head injury, fatality at the scene, family members also injured, death of a family member and length of stay (total and intensive care unit).

Ratio measures (OR) will be used to measure the effect of the main outcome. The effect estimates and a CI or p value will be presented when possible. For studies reporting on the same outcome, the magnitude of variation or heterogeneity between studies will be measured by the index of heterogeneity (I^2^ and its CIs). I^2^ values of 25%, 50% and 75% are assumed to represent low, medium and high heterogeneity, respectively. χ^2^ will determine the significance of the heterogeneity for Q statistics. If high heterogeneity is identified in the preliminary stages of meta-analysis, we will report individual study results (including forest plotting for visualisation) without reporting a pooled effect estimate.

### Patient and public involvement

No specific patient and public activities were undertaken for this review. However, as part of a broader and ongoing paediatric trauma research stream, the Bristol Young Person’s Advisory Group has been consulted.

## Ethics and dissemination

Ethical review is not required as no original data will be collected. Results will be disseminated through peer-reviewed publications and at academic conferences.

## Supplementary material

10.1136/bmjopen-2025-101971 online supplemental file 1
