# Supplementary material for: Psychological outcomes in paediatric major trauma patients who require invasive management: protocol for a systematic review and meta-analysis
Source: BMJ Open. 2025 Aug 19;15(8):e101971. doi: 10.1136/bmjopen-2025-101971 (PMC12366560; doi:10.1136/bmjopen-2025-101971)
Supplement: online supplemental file 1 [file bmjopen-15-8-s001.docx]

**Appendices**

**Appendix 1. Provisional testing of the search strategy prior to starting the systematic review**

**Ovid MEDLINE(R) and Epub Ahead of Print, In-Process, In-Data-Review & Other Non-Indexed Citations, Daily and Versions <1946 to February 27, 2025>**

1 exp Pediatrics/ 64899

2 adolescent/ or child/ or child, preschool/ or infant/ or infant, newborn/ 4139558

3 (paediatric* or pediatric* or child* or toddler* or infant* or neonat* or newborn* or preschool* or "pre-school*" or adolescen* or teen*).ti,ab,kw,kf. 2856325

4 1 or 2 or 3 4977821

5 General Surgery/ 41340

6 Orthopedics/ 25127

7 (surg* or orthop* or operat* or "major trauma*" or "major injur*").ti,ab,kw,kf. 3637858

8 5 or 6 or 7 3653723

9 Mental Disorders/ 185325

10 Adjustment Disorders/ 4350

11 Depression/ 166938

12 Stress Disorders, Post-Traumatic/ 45018

13 Anxiety/ or Anxiety Disorders/ 151941

14 (depress* or anxiety or PTSD or posttraumatic or "post traumatic" or "stress disorder*").ti,ab,kw,kf. 837658

15 ((mental or psycholog* or psychiatric or adjustment) adj (disorder* or disease* or illness* or health)).ti,ab,kw,kf. 422405

16 9 or 10 or 11 or 12 or 13 or 14 or 15 1253291

17 Risk Factors/ 1021244

18 Epidemiology/ 12645

19 Prevalence/ 367209

20 Incidence/ 317378

21 ("risk factor*" or prevalen* or incidence or epidemiolog*).ti,ab,kw,kf. 2960064

22 17 or 18 or 19 or 20 or 21 3539927

23 4 and 8 and 16 and 22 4436

**Embase <1974 to 2025 February 26>**

1 exp pediatrics/ 133231

2 child/ or hospitalized child/ or infant/ or preschool child/ or school child/ or toddler/ or newborn/ or adolescent/ or hospitalized adolescent/      4189145

3 (paediatric* or pediatric* or child* or toddler* or infant* or neonat* or newborn* or preschool* or "pre-school*" or adolescen* or teen*).ti,ab,kw,kf. 3544036

4 1 or 2 or 3 5178288

5 major injury/ or surgery/ or orthopedics/ 899351

6 (surg* or orthop* or operat* or "major trauma*" or "major injur*").ti,ab,kw,kf. 4713016

7 mental disease/ or adjustment disorder/ or depression/ or major depression/ or posttraumatic stress disorder/ 918246

8 (depress* or anxiety or PTSD or posttraumatic or "post traumatic" or "stress disorder*").ti,ab,kw,kf. 1108649

9 ((mental or psycholog* or psychiatric or adjustment) adj (disorder* or disease* or illness* or health)).ti,ab,kw,kf. 520720

10 5 or 6 4925803

11 7 or 8 or 9 1714718

12 risk factor/ or epidemiology/ or prevalence/ or incidence/ 3042971

13 ("risk factor*" or prevalen* or incidence or epidemiolog*).ti,ab,kw,kf. 4149626

14 12 or 13 5086774

15 4 and 10 and 11 and 14 6111

**PsycInfo via Ebscohost**

DE "Pediatrics" OR TI ( paediatric* or pediatric* or child* or toddler* or infant* or neonat* or newborn* or preschool* or "pre-school*" or adolescen* or teen* ) OR AB ( paediatric* or pediatric* or child* or toddler* or infant* or neonat* or newborn* or preschool* or "pre-school*" or adolescen* or teen* )

AND

DE "Surgery" OR TI ( surg* or orthop* or operat* or "major trauma*" or "major injur*" ) OR AB ( surg* or orthop* or operat* or "major trauma*" or "major injur*" )

AND

DE "Mental Disorders"  OR  DE "Adjustment Disorders" OR  DE "Anxiety" OR DE "Anxiety Disorders" OR  DE "Major Depression" OR  DE "Posttraumatic Stress Disorder" OR TI ( depress* or anxiety or PTSD or posttraumatic or "post traumatic" or "stress disorder*" ) OR AB ( depress* or anxiety or PTSD or posttraumatic or "post traumatic" or "stress disorder*" )  OR TI ( (mental or psycholog* or psychiatric or adjustment) N1 (disorder* or disease* or illness* or health) ) OR AB ( (mental or psycholog* or psychiatric or adjustment) N1 (disorder* or disease* or illness* or health) )

AND

DE "Risk Factors" OR  DE "Epidemiology" OR TI ( "risk factor*" or prevalen* or incidence or epidemiolog* ) OR AB ( "risk factor*" or prevalen* or incidence or epidemiolog* )

**Cinahl via Ebscohost**

(MH "Pediatrics+") OR (MH "Child") OR (MH "Child, Hospitalized") OR (MH "Infant, Newborn") OR (MH "Infant") OR (MH "Child, Preschool") OR (MH "Adolescent, Hospitalized")

AND

(MH "Surgery, Operative") OR (MH "Orthopedics")  OR TI ( surg* or orthop* or operat* or "major trauma*" or "major injur*" ) OR AB ( surg* or orthop* or operat* or "major trauma*" or "major injur*" )

AND

(MH "Mental Disorders") OR (MH "Adjustment Disorders") OR (MH "Depression") OR (MH "Anxiety") OR (MH "Stress Disorders, Post-Traumatic")  OR OR TI ( depress* or anxiety or PTSD or posttraumatic or "post traumatic" or "stress disorder*" ) OR AB ( depress* or anxiety or PTSD or posttraumatic or "post traumatic" or "stress disorder*" )  OR TI ( (mental or psycholog* or psychiatric or adjustment) N1 (disorder* or disease* or illness* or health) ) OR AB ( (mental or psycholog* or psychiatric or adjustment) N1 (disorder* or disease* or illness* or health) )

AND

(MH "Risk Factors") OR (MH "Epidemiology") OR (MH "Prevalence") OR (MH "Incidence")  OR TI ( "risk factor*" or prevalen* or incidence or epidemiolog* ) OR AB ( "risk factor*" or prevalen* or incidence or epidemiolog* )

**Provisional results**

| **Database** | **Results 28/02/2025** |
| --- | --- |
| Medline | 4436 |
| Embase | 6111 |
| PsycInfo | 1123 |
| Cinahl | 832 |
| After deduplication | 8471 |
