# Supplementary material for: Psychological outcomes in paediatric major trauma patients who require invasive management: protocol for a systematic review and meta-analysis
Source: BMJ Open. 2025 Aug 19;15(8):e101971. doi: 10.1136/bmjopen-2025-101971 (PMC12366560; doi:10.1136/bmjopen-2025-101971)
Supplement: Supplementary file 2 [file bmjopen-2025-101971.reviewer_comments.pdf]

PEER REVIEW HISTORY

BMJ Open publishes all reviews undertaken for accepted manuscripts. Reviewers are asked to complete a checklist review form and are provided with free text boxes to elaborate on their assessment. These free text comments are reproduced below.

ARTICLE DETAILS

Title (Provisional)

Psychological Outcomes in Paediatric Major Trauma Patients Who Require Invasive Management: Protocol for a Systematic Review and Meta-Analysis

Authors

Hibberd, Owen; Thomas, Caroline E; Gentle, Sarah; Angus, Sandi; Karageorgos, Spyridon; Phillips, Veronica; Thomas, Stephen H.

VERSION 1 - REVIEW

Reviewer **1**

Name **Mullen, Stephen**

Affiliation **Royal Belfast Children's Hospital, Paediatric Emergency Department, University Hospitals of Wales,**

Date **05-Apr-2025**

COI **None**

Many thanks for allowing me to review this protocol. The topic is interesting and one that requires further input to address the gap in knowledge so I commend the authors for undertaking this piece of work.

A few questions:

1) why group IR/surgery together

2) how are you balancing variables: HI vs non HI, family member involved in accident and died vs not, that may have an impact

3) I would imagine the intervention group will have a higher ISS, longer LoS (total and ICU) than your control. How are you adjusting for this ie if you have more seriously injured with a longer length of stay you will probably be more likely to have long term psych issues and the findings may not solely be related to the surgery/IR procedure

---

Reviewer **2**

Name **Enomoto, Yuki**

**Affiliation**	**University of Tsukuba**
**Date**	**31-Jul-2025**
**COI**	**None**

This paper describes a plan for a systematic review on mental health in children after major trauma. This is a very important research topic, and the results will be interesting to many readers. The study plan is well-written. I have two minor comments to make the protocol clearer.

1. Please state if you will only search for studies written in English or if you will include studies in other languages as well. It is important to mention this in the methods section.

2. Your outcome is a "new diagnosis of PTSD/anxiety/depression or adjustment disorder." Please explain how you will handle patients who already had a mental health condition before their injury. Will you include or exclude them?

VERSION 1 - AUTHOR RESPONSE

Reviewer: 1

Dr. Stephen Mullen, Royal Belfast Children's Hospital, Queen's University Belfast

Comments to the Author: Many thanks for allowing me to review this protocol. The topic is interesting and one that requires further input to address the gap in knowledge so I commend the authors for undertaking this piece of work.
Thank you, Dr Mullen. We want to thank you for your review and comments. We believe these have greatly enhanced the quality of this protocol and subsequent review. A few questions:
1) why group IR/surgery together
Thank you. This is a good question which prompted further reflection from us. We have now updated this to extract data on IR and surgery separately. These will be analysed as both individual and combined variables.
Operation and Interventional Radiology will be analysed separately, but if these cannot be separated in the data extraction, they will be combined as 'invasive management'.

2) how are you balancing variables: HI vs non HI, family member involved in accident and died vs not, that may have an impact
Thank you. This is a very insightful comment, and we agree these are highly relevant. We have included these as variables for extraction and analysis when they are present.
As per standard recommendations from Cochrane, we will plan to execute subgroup-focused analyses, such as meta-regression, if we can accumulate approximately ten studies. If there is sufficient data, a meta-regression will include other potential effect modifiers such as ISS, head injury, fatality at the scene, family members also injured, death of a family member, and length of stay (total and ICU).

3) I would imagine the intervention group will have a higher ISS, longer LoS (total and ICU) than your control. How are you adjusting for this ie if you have more seriously injured with a longer length of stay you will probably be more likely to have long term psych issues and the findings may not solely be related to the surgery/IR procedure
Thank you. We again thank you for your insightful comments. We have updated the analysis plan to include a meta-regression that features relevant potential effect modifiers.
As per standard recommendations from Cochrane, we will plan to execute subgroup-focused analyses, such as meta-regression, if we can accumulate approximately ten studies. If there is sufficient data, a meta-regression will include other potential effect modifiers such as ISS, head injury, fatality at the scene, family members also injured, death of a family member, and length of stay (total and ICU).

Reviewer: 2
Dr. Yuki Enomoto, University of Tsukuba
Thank you, Dr Enomoto. We want to thank you for your review and comments. We believe these have greatly enhanced the quality of this protocol and subsequent review.
Comments to the Author: This paper describes a plan for a systematic review on mental health in children after major trauma. This is a very important research topic, and the results will be interesting to many readers. The study plan is well-written. I have two minor comments to make the protocol clearer. 1. Please state if you will only search for studies written in English or if you will include studies in other languages as well. It is important to mention this in the methods section.

Thank you. Indeed, we initially planned to only search for studies written in English. However, this insightful comment prompted us to consider how proportionally more paediatric trauma may be seen in countries which would not necessarily publish in the English language.

Studies published in any language that meet the inclusion criteria will be included. If the study is published in a language other than English, it will be included if sufficient data can be extracted using open-access translation software such as *'Google Translate'.*
2. Your outcome is a "new diagnosis of PTSD/anxiety/depression or adjustment disorder." Please explain how you will handle patients who already had a mental health condition before their injury. Will you include or exclude them?
Thank you. We appreciate this helpful comment and agree that it is an important consideration. For the purposes of this review, we included this in the descriptive analysis. This aims to describe the overall cohort, and since we hypothesise that the cohort of patients with a pre-existing mental health condition may require further consideration for psychological support.
A descriptive analysis will be conducted. The descriptive analysis will also include a description of injured patients with a previous mental health diagnosis to describe the overall cohort.

Reviewer: 1 If you have selected 'Yes' above, please provide details of any competing interests.: NA Reviewer: 2 If you have selected 'Yes' above, please provide details of any competing interests.: Not applicable
